# Human T-Lymphotropic Virus-1/2 Infection in Central Brazil Prisons: A Multicenter Study

**DOI:** 10.3389/fmicb.2021.740245

**Published:** 2022-01-21

**Authors:** Larissa Melo Bandeira, Marco Antonio Moreira Puga, Julio Croda, Maurício Antonio Pompílio, Carolina Amianti, Grazielli Rocha de Rezende, Ana Rita Coimbra Motta-Castro

**Affiliations:** ^1^Federal University of Mato Grosso do Sul, Campo Grande, Brazil; ^2^Fiocruz Mato Grosso do Sul, Fundação Oswaldo Cruz, Ministério da Saúde, Campo Grande, Brazil

**Keywords:** human T-lymphotropic virus, prevalence, prisoners, high-risk population, Brazil

## Abstract

Human T-lymphotropic virus (HTLV) infection is endemic in some group populations. Prisoners are at high risk of acquiring HTLV infection mainly due to the environment of closed penal institutions, socioeconomic conditions, and risk behaviors. This study aimed to investigate the prevalence, the occurrence of new cases of HTLV infection after a 1-year follow-up, and circulating subtypes of HTLV-1 among prisoners from twelve closed prisons in Mato Grosso do Sul state, Central Brazil. A total of 1,507 prisoners were randomly enrolled in the cross-sectional study. Out of the 1,507 participants, 1,000 prisoners, susceptible to HTLV infection, were included in the prospective cohort study. In the cross-sectional study, serological evidence of HTLV infection was 0.4% (CI 95%: 0.1-0.7), tested for anti-HTLV antibodies by enzyme-linked immunosorbent assay (ELISA). Three samples were positive for HTLV-1, two samples were positive for HTLV-2, and 1 sample was indeterminate by the Western blot method. The presence of HTLV proviral DNA was detected in all positive samples by amplification of the HTLV *tax* gene through nested-PCR. Phylogenetic analysis showed that HTLV-1 samples belonged to the Cosmopolitan subtype Transcontinental subgroup. From a cohort of 1,000 individuals, no new case of HTLV infection was detected. Although the prevalence rate of HTLV infection found in this study was similar to that observed in the Brazilian general population, the lack of access to preventive interventions and harm reduction measures all contribute to increasing the risk of HTLV transmission and acquisition among this key and vulnerable population.

## Introduction

Inmates represent a highly vulnerable population to several infectious diseases compared with the general population, particularly blood, and sexually transmittable infections (STI) (Massoglia and Remster, 2019). Use of intravenous drug use, needle sharing, sex work, tattoos, and piercing, or men having sex with other men are behaviors associated with a higher risk of acquiring these infections during the period before incarceration as well as during imprisonment (Augusto et al., 2017).

Brazil has the third-largest prison population in the world, with approximately 702,000 inmates in 2020 (Puga et al., 2019; DEPEN, 2020). Heath care, prevention, diagnosis, and treatment programs for infectious diseases are limited inside the correctional institutions (Fialho et al., 2008). The state of Mato Grosso do Sul (MS) in Central Brazil has approximately 17,600 inmates, it was the highest rate of incarceration in the country in 2017 (Puga et al., 2019; DEPEN, 2020).

The Human T-lymphotropic virus (HTLV) is a retrovirus that has been found throughout the world (Gessain and Cassar, 2012). In Brazil, the prevalence of HTLV-1/2 infection varies according to the region and the population group studied. The prevalence of HTLV-1/2 in blood donors of Brazil ranges from 0.09 to 0.48% (Catalan-Soares et al., 2005; Pinto et al., 2012; Ribeiro et al., 2018; Pessoni et al., 2019). A systematic review study estimated a prevalence of HTLV-1 in Brazilian pregnant women of 0.32%, while HTLV-2 was 0.04% (Vieira et al., 2021). This virus is transmitted via unprotected sexual intercourse, exposure to the infected blood, or from infected mother to child mainly through breastfeeding (Nunes et al., 2017).

Considering the relevance of HTLV-1/2 infection and limited data to this infection among the prison population, this study aimed to investigate the prevalence, the occurrence of new cases of HTLV infection after a 1-year follow-up, and circulating subtypes of HTLV-1 infection among prisoners from twelve closed penal institutions in Central Brazil.

## Materials and Methods

### Study Population

**FIGURE 1:**
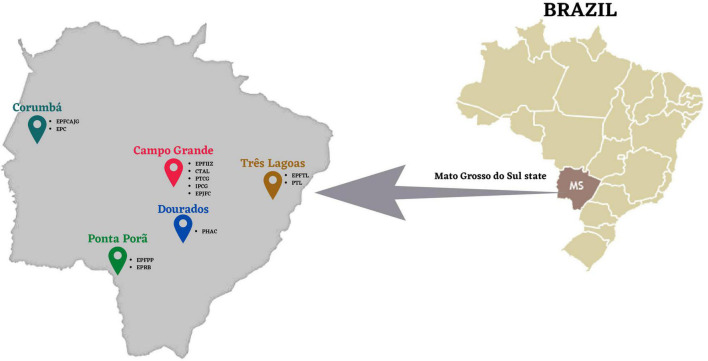
The geographic location of study prisons, Mato Grosso do Sul State, Brazil (Adapted from Puga et al., 2017).

The study population consisted of inmates from 12 of the 21 closed penal institutions of five different cities of Mato Grosso do Sul State, Central Brazil, as follows: Campo Grande, Corumbá, Ponta Porã, Dourados, and Três Lagoas (Figure 1). Participants underwent an interview via a standard questionnaire containing information about personal, demographic data, and other variables of interest such as a history of injection drug use and sexual practices. All subjects gave their written informed consent to participate in the study. Participation in the study was voluntary, confidential, and no compensation was provided. Blood samples were collected from all subjects to perform serological tests.

According to the sample size calculation based on the estimated prevalence for anti-HTLV-1/2 of 1.05% (mean) for anti-HTLV found among Brazilian inmate studies (Broutet et al., 1996; Catalan-Soares et al., 2000; Fialho et al., 2008), a significance level of 99% (α < 0.05), and 1% of alpha-type error the present study should include at least 582 prisoners. Then, between March 2015 and March 2018, a random selection of 1,507 from 3,368 participants of the previous cross-sectional study was performed by individuals stratified proportionally by each prison using Epi-Info 6.04 software (CDC, Atlanta, GA, United States) to estimate the prevalence rate of HTLV infection.

To investigate the occurrence of new cases of HTLV infection after a 1-year follow-up, 1,000 from the 1,507 participants were included in the prospective cohort study. The inclusion criteria were (a) having participated in the prevalence study; (b) not leaving prisons (for any reason) during this period; (c) having samples stored in sufficient quantity to perform the analyses proposed; and (d) being susceptible to HTLV infection (anti-HTLV-1/2 negative).

### Serological Tests

The selected serum samples were screened by an enzyme-linked immunosorbent assay (ELISA) commercial kit for the presence of anti-HTLV-1/2 antibodies (GOLD ELISA HTLV-I/II – REM), following the manufacturer’s instructions. Positive samples were repeatedly tested and confirmed by HTLV-1/2 Western Blot (WB) assay (MP Diagnostics HTLV BLOT 2.4–Singapore).

The samples reactive by screening and positive by WB were considered positive for HTLV-1 or 2 infections. The selected serum samples were also tested by ELISA method for anti-HIV-1/2 (Murex HIV-1.2.0, DiaSorin, Italy), anti-HCV (ELISA–Murex Diagnostics, United Kingdom), and for the presence of HBV serological markers (HBsAg, anti-HBs, and total anti-HBc) (ELISA, DiaSorin S.p.A) as described before by Sgarbi et al. (2015), and Puga et al. (2017) and Rezende et al. (2020), respectively.

### Human T-Lymphotropic Virus-1/2 Molecular Characterization

Reactive samples (anti-HTLV-1/2 positive) were also confirmed by nested polymerase chain reaction (PCR). To this end, a whole blood collection from anti-HTLV positive individuals was performed and DNA was extracted from these samples using the QIAamp DNA Blood mini kit (QIAgen), according to the manufacturer’s instructions. The amplification of a 218 bp fragment of the HTLV *Tax* gene of both HTLV-1 and -2 was performed to confirm the HTLV-1/2 infection as previously described (Bandeira et al., 2015). Furthermore, the amplification of a 672 bp fragment of the HTLV-1 5′LTR region was performed on the same samples by nested PCR for confirmation of the HTLV type 1 infection, specifically, as previously reported (Bandeira et al., 2015), and to sequencing analysis. The amplicons were purified using PureLink Quick PCR Purification Kit (Invitrogen, Lithuania), according to the manufacturer’s instructions. The fragments were sequenced using BigDye Terminator Cycle Sequencing Ready Reaction Kit and ABI 1373 (Applied Biosystems, Foster City, CA, United States) using Sanger’s method. After nucleotide sequencing, the isolates from the HTLV-1 5′LTR region were subjected to an analysis in the BLAST (Basic Local Alignment Search Tool). The HTLV-2 LTR amplification was not performed since financial resources for specific reagents were not available at the moment.

All anti-HTLV-1 infected individuals were recalled for a new blood collection to confirm the result. On this occasion, all anti-HTLV-1 infected individuals were submitted to the first clinical screening by an infectious disease specialist of our group. Hematologic tests and clinical manifestations of this disease such as slow and progressive paraplegia of the lower extremities, spasticity, hyperreflexia, bladder and bowel dysfunction, lower back pain, and sphincter disorders were investigated.

This study was approved by the Federal University of Mato Grosso do Sul Ethics Committee on Human Research, under protocol number 1.250.132, CAAE: 49361715.8.0000.0021.

### Statistical Analysis

Statistical analyses were performed using STATA 13.0 software (Stata Corporation, College Station, TX, United States). The prevalence of HTLV infection, estimated with 95% confidence intervals, and the categorical variables were presented by absolute and percentage frequency. The chi-square test was used to evaluate differences between proportions. Continuous variables were expressed as mean, standard deviation, median, and range. The sample size was calculated using BioEstat 5.0.

## Results

A total of 1,507 prisoners were included in the cross-sectional study. The median age of participants was 29 ± 10 years (range 18-76), including 1,307 males (86.73%) and 200 females (13.27%). The majority reported less than 9 years of formal education (73.39%) and was from Mato Grosso do Sul State (67.00%). Regarding ethnicity, most of them declared themselves as multiracial (52.60%).

Low frequency (1.16%) of inmates reported a history of illicit injection drug use (IDU). History of drug use such as marihuana, cocaine, and crack was reported by 29.40%, 18.50%, and 4.20% of prisoners, respectively. Sharing sharp objects was reported by 3.30% of the participants and was more frequent among males (76.00%). History of blood transfusion was informed by 12.64% of subjects, of whom 33 reported that received a blood transfusion before 1993 when the screening for anti-HTLV became mandatory in Brazilian blood banks. The presence of tattoos was reported by 66.95%.

Regarding risk characteristics related to sexual transmission, prisoners reported a high frequency of irregular condom use (67.18%). History of STI was reported by 12.32% of participants. Most of the prisoners declared themselves as heterosexual (97.46%), however, 8.00% reported that they had already had homosexual intercourse. 51.70% of individuals reported less than 10 sexual partners in the past 5 years. Anti-HIV and anti-HCV were detected in 22 (1.46%) and 37 (2.46%) samples of the 1,507 prisoners. Table 1 shows the characteristics of the cross-sectional and prospective cohort studied population.

**TABLE 1:** Characteristics of 1,507 inmates of the cross-sectional study and of 1,000 inmates of the cohort study.

Characteristics	Cross-sectional study (*N* = 1,507)		Prospective cohort study (*N* = 1,000)	
	N	%	N	%
**Age (years)^a^**				
18–24	358	24.22	183	18.47
25–39	834	56.43	608	61.35
40–59	271	18.34	191	19.27
≥ 60	15	1.01	9	0.91
**Gender**				
Female	200	13.27	130	13.00
Male	1.307	86.73	870	87.00
**Education level (years)^a^**				
≤ 9	1073	73.39	743	75.13
> 9	389	26.61	246	24.87
**Marital status^a^**				
Single	633	43.00	449	46.82
Married	469	31.86	399	41.61
Divorced/widowed	370	25.14	111	11.57
**Ethnicity^a^**				
White	488	32.95	324	33.68
Multiracial	779	52.60	500	51.97
Black	164	11.07	108	11.23
Asian	34	2.30	17	1.77
Indigenous	16	1.08	13	1.35
**City of imprisonment**				
Campo Grande	789	52.36	492	49.20
Corumbá	161	10.68	114	11.40
Dourados	270	17.92	225	22.50
Ponta Porã	148	9.82	94	9.40
Três Lagoas	139	9.22	75	7.50
**History of Sexually transmitted infection^a^**				
No	1246	87.68	953	96.55
Yes	175	12.32	34	3.45
**Homosexual relation^a^**				
No	1333	92.00	918	93.67
Yes	116	8.00	62	6.33
**Condom use ^a^**				
Always	490	32.82	142	22.94
Sometimes/Never	1003	67.18	477	77.06
**History of injectable drug use ^a^**				
No	1443	98.84	994	99.70
Yes	17	1.16	3	0.30
**Injectable drug use in prison^a^**				
No	154	98.09	939	99.89
Yes	3	1.91	1	0.11
**History of Blood Transfusion^a^**				
No	1292	87.36	773	78.32
Yes	187	12.64	214	21.68
**Blood Transfusion over the last year^a^**				
No	–	–	980	98.59
Yes	–	–	14	1.41
**Tattoo *^a^***				
No	497	33.05	–	–
Yes	1007	66.95	–	–
**Tattoo over the last year^a^**				
No	–	–	860	86.17
Yes	–	–	138	13.83
**Needle sharing *^a^***				
No	1447	96.66	–	–
Yes	50	3.34	–	–
**Needle sharing over the last year^a^**				
No	–	–	982	98.69
Yes	–	–	13	1.31
**Anti-HIV**				
Negative	1485	98.54	982	98.20
Positive	22	1.46	18	1.80
**Anti-HCV**				
Negative	1470	97.54	992	99.20
Positive	37	2.46	8	0.80
**Anti-Hbc**				
Negative	1365	90.58	960	96.00
Positive	142	9.42	40	4.00

***^a^** The total represents the number of individuals who answered the question. Percentages were calculated excluding missing data.*

**FIGURE 2:**
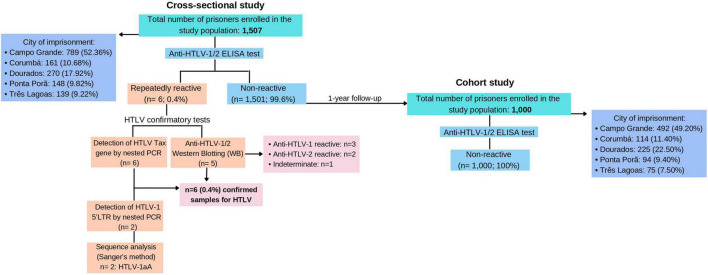
Flow chart of the screening process for the detection of HTLV infection. Abbreviations: EPFCAJG – Estabelecimento Penal Feminino Carlos Alberto Jonas Giordano, EPC – Estabelecimento Penal de Corumbá, EPFIIZ – Estabelecimento Penal Feminino Irmã Irma Zorzi, CTAL – Centro de Triagem Anízio Lima, PTCG – Presídio de Trânsito de Campo Grande, IPCG – Instituto Penal de Campo Grande, EPJFC – Estabelecimento Penal Jair Ferreira de Carvalho, PHAC – Penitenciária Harry Amorim Costa, EPFPP – Estabelecimento Penal Feminino de Ponta Porã, EPRB – Estabelecimento Penal Ricardo Brandão, EPFTL – Estabelecimento Penal Feminino de Três Lagoas, PTL – Penitenciária de Três Lagoas.

Anti-HTLV was detected in six prisoners [0.40% (CI 95%: 0.1-0.7)] in the cross-sectional study (Figure 2). All ELISA repeatedly reactive samples were further tested by WB and PCR assay. No double infection was found. Five samples confirmed anti-HTLV reactivity with complete profiles in the WB test: three samples were positive for HTLV-1, two samples were positive for HTLV-2, and 1 sample was indeterminate HTLV.

The presence of HTLV proviral DNA was detected in all positive samples (*n* = 6) by amplification of the HTLV *Tax* gene through nested PCR. Out of 6 confirmed samples, only 2 (33.33%) amplified HTLV-1 5′LTR region and were successfully sequenced by the Sanger method. The nucleotide sequencing of the isolates from the HTLV-1 5′LTR region was subjected to an analysis in the BLAST and the 2 isolates were classified as Cosmopolitan (1a) subtype and Transcontinental (A) subgroup. Nucleotide sequences were registered into the GenBank database under accession numbers OK247616 and OK247617.

Table 2 shows sociodemographic and risk behavior characteristics of anti-HTLV positive prisoners of the cross-sectional study. All of them were males and reported less than 9 years of formal education. Concerning the distribution of anti-HTLV by age, the HTLV infected prisoners were over 29 years old, ranging from 29 to 62 years old. Regarding ethnicity, most of them declared themselves as multiracial. Unsafe sexual behavior was recorded in 83.33% of anti-HTLV seropositive subjects. Most of the HTLV infection cases were among prisoners incarcerated in Campo Grande prisons (4/6), and 3 of these were from the same prison (EPJFC) and pavilion (ID-3270, ID-3417, and ID-3484). Two HTLV infection cases were among prisoners incarcerated in the same Três Lagoas prison (PTL). The median time of incarceration of the positive ones was 292.16 months, while the anti-HTLV negative group was 99 months. Because of the low number of infected anti-HTLV individuals found in this study, our ability to detect associated risk factors was limited.

**TABLE 2:** Sociodemographic and risk behavior characteristics of the six anti-HTLV infected prisoners of the cross-sectional study.

*Characteristics*	ID-452	ID-2545	ID-3417	ID-3270	ID-3484	ID-573
HTLV	HTLV-1aA	HTLV-1aA	HTLV-1	HTLV-2	HTLV-2	HTLV
Age (years)	33	62	55	46	29	44
Marital status	Married	Married	Married	Single	Single	Single
Gender	Male	Male	Male	Male	Male	Male
Ethnicity	Multiracial	Black	Multiracial	Multiracial	White	Multiracial
Naturality	MS	MS	RN	SP	MS	MS
City of imprisonment	Três Lagoas	Campo Grande	Campo Grande	Campo Grande	Campo Grande	Três Lagoas
Prison	PTL	IPCG	EPJFC	EPJFC	EPJFC	PTL
IDU history	No	No	Yes	Yes	No	No
Blood transfusion before 1993	No	No	No	No	No	No
Condom use	Always	Sometimes	Never	Sometimes	Never	Sometimes
Sexual preference	Heterosexual	Heterosexual	Heterosexual	Heterosexual	Heterosexual	Heterosexual
Previous incarceration	Yes	Yes	Yes	Yes	No	No
Time in prison (months)	12	324	NA	NA	156	1
Anti-HIV positive	Negative	Negative	Negative	Negative	Negative	Positive
Anti-HCV positive	Negative	Positive	Positive	Positive	Negative	Negative
HBsAg positive	Negative	Negative	Negative	Negative	Negative	Negative
Total anti-HBc positive	Positive	Negative	Negative	Positive	Negative	Negative
Active tuberculosis	Negative	Negative	Negative	Negative	Negative	Negative

*ID-identification number of the participant sample, IDU-Injection drug use, MS-Mato Grosso do Sul state, RN-Rio Grande do Norte state, SP-São Paulo state NA: data not available.*

Those participants infected with HTLV-1 were subsequently referred to a clinical assessment by the prison infectious disease specialist. After clinical evaluations, none of the anti-HTLV-1 positive subjects showed clinical signs compatible with HAM/TSP or ATL. Only ID-3270 had already been tested for anti-HTLV and had a positive HTLV-2 result in his medical record. He reported injecting drug use with needle sharing, was breastfed for approximately 1 year, and had unsafe sexual behavior. It is noteworthy that half of the anti-HTLV positive individuals were co-infected with HCV and 1 of them was co-infected with HIV. There was a significant difference of positivity for anti-HCV (8.11% *vs.* 0.20%; *p* < 0.001) between anti-HTLV positive and non-HTLV-positive prisoners.

Out of the 1,507 participants included in the cross-sectional study, 1,000 were included in the cohort study. Among them, no new case of anti-HTLV infection was detected. Low frequency was found for these variables among the studied population in 1-year-follow-up: tattooing over the last year (13.83%), sharing needle over the last year (1.30%), injecting drug use in prison (0.11%), and history of blood transfusion over the last year (1.41%).

## Discussion

This is the first survey on HTLV-1/2 infection prevalence carried out among inmates in Central Brazil, involving the five major closed penal institutions of Mato Grosso do Sul State (DEPEN, 2020). The high-risk behavior to which this vulnerable population is subjected and the critical conditions of Brazilian penitentiaries motivated us to conduct this study. The study population consisted of inmates who participated in a previous cross-sectional study that included individuals from 12 of the 21 closed penal institutions of five different cities of Mato Grosso do Sul State, Central Brazil (Campo Grande, Corumbá, Ponta Porã, Dourados, and Três Lagoas) (Puga et al., 2017). Stored samples from the previous study were used.

The HTLV prevalence found in this study [0.40% (CI 95%: 0.1–0.7)] is similar to the prevalence observed in the general population from Mato Grosso do Sul such as blood donors and pregnant, 0.2% and 0.1%, respectively (Freitas, 2002; Dal Frabbro et al., 2008). In addition, our results are consistent with a prior national study conducted among prisoners from Fortaleza (0.47%), a city of Northeast Brazil (Broutet et al., 1996). However, Catalan-Soares et al. found a seroprevalence of 1.6%, but this study included a considerably smaller number of participants. Although it has important implications for public health, it could not be considered representative of all prisons in Brazil (Catalan-Soares et al., 2000). The Prevalence of HTLV-1/2 infection in incarcerated adolescents from Salvador (1.09%) also was higher compared with our study (Fialho et al., 2008), which can be explained by the fact that HTLV infection is prevalent in this area of Brazil.

Additionally, when compared to international studies, the prevalence of HTLV infection found in our study was lower than that reported among imprisoned immigrants from Northern Italy that found HTLV infection prevalence of 1.1%, corresponding to 1.4% of HTLV-1 infection and 0.8% of HTLV-2 infection (Ansaldi et al., 2003). A study in a Danish medium-security prison for males found no cases of HTLV infection (Christensen et al., 2000).

Few studies assessed higher HTLV infection prevalence in prisoners, most of them included inmates with a history of injection drug use or with HIV infection, which may justify a higher number of infectious diseases in these studies compared to our findings (Prasetyo et al., 2013; Augusto et al., 2017).

The presence of HTLV-2 infection among prisoners studied was an important observation coming out of this survey. Most cases of HTLV-2 infection described in Brazil have occurred among people who inject drugs (PWID) and the indigenous population (Braço et al., 2019; Oliveira-Filho et al., 2019). There were few PWID in our sample (1.2%; 17/1,460), but if we considered only the individuals who reported injecting drug use, the rate of HTLV infection would be 11.8% (2/17). It is known that the PWID is engaging in high-risk injection practices such as needle/syringe sharing, increasing the risk of acquiring infectious diseases such as HTLV, HIV, HCV, and HBV infections (de la Fuente et al., 2006). It was observed that half of the anti-HTLV positive individuals were co-infected with HCV, and two of them reported injection drug use history. In the HCV prevalence study conducted in the same population, from which this study departed, injection drug use was associated with HCV exposure in male prisoners (Puga et al., 2017). In addition, the serological evidence of past HBV infection (33.3%) found in anti-HTLV positive individuals of this study was 3.4 times higher than in the prisoners enrolled in the previous study (9.8%) conducted among 3,368 inmates (Rezende et al., 2020). Moreover, most anti-HTLV positive participants were still susceptible to HBV infection (66.7%). These results and the comparable rates found in other studies among inmates suggest that prisoners still have a greater vulnerability to HBV acquisition (Fialho et al., 2008; Rezende et al., 2020).

It was observed that only one anti-HTLV positive individual was co-infected with HIV. Although there is strong evidence that HTLV and HIV may be transmitted to susceptible individuals by the same routes, in this case, risk behaviors such as a history of STI, injecting drug use, multiple sexual partners, and sharing needles were not reported by him. However, he reported irregular condom use, non-injected drug use, and a history of treating tuberculosis. Conclusions regarding routes of transmission cannot be drawn from this single case of HTLV/HIV co-infection (Osti et al., 1998; Prasetyo et al., 2013; Kozlowski et al., 2016).

The distribution of HTLV-1/2 infection by gender differs from previous reports which found a higher prevalence of HTLV infection in female prisoners, possibly reflecting the high-risk behavior observed among male prisoners [multiple sexual partners, sexual risk practices, and history of injection drug use (Ansaldi et al., 2003; Augusto et al., 2017)].

One sample (ID-573) was HTLV indeterminate in the western blot test, but anti-HTLV was detected by ELISA method and the presence of HTLV proviral DNA was detected by amplification of the HTLV *Tax* gene through nested-PCR. The finding of indeterminate results in the WB for HTLV and positive by PCR has already been reported in other studies (Broutet et al., 1996; Osti et al., 1998; Ji et al., 2020; Halbrook et al., 2021). About this phenomenon, one hypothesis that we can consider is a longer time for seroconversion that can be associated with low antigenic stimulation due to the defective proviruses (Cánepa et al., 2015; Kuramitsu et al., 2017; Gomes et al., 2020). Therefore, this sample was considered HTLV positive due to confirmation by nested-PCR.

The HTLV transmission could occur through blood transfusion until 1993 when serological screening for HTLV-1/2 became mandatory in Brazilian blood banks. History of blood transfusion was found in one-third of HTLV infected individuals (ID-452, ID-3270), but it occurred after 1993. However, both individuals reported other risk factors, such as irregular condom use (ID-452, ID-3270) and injecting drug use (ID-3270).

It is known that a greater number of sexual partners is a high-risk behavior factor for acquiring sexually transmitted infections, such as HTLV (Castro et al., 2018). However, the majority of the cross-sectional study population reported having had sexual intercourse with less than 10 people in the past 5 years. This behavior was also observed in HTLV positive individuals, who reported only one sexual partner in the last 5 years, except for ID-452 who reported 5 sexual partners in this period.

The positive anti-HTLV-1 samples ID-452 and ID-2545 were classified as belonging to the Transcontinental (A) subgroup of the Cosmopolitan (a) subtype. HTLV-1aA is considered the most prevalent in Brazil (Ishak et al., 2020).

Those participants infected with HTLV-1 were subsequently referred to a clinical assessment by the prison infectious disease physician and an investigation of symptoms was carried out. No sensory, motor, and hematological changes were observed. Furthermore, no deficits were observed in the general and neurological examination.

Although new cases of HCV and HIV infections were found by our group in cohort studies conducted in the same population (Sgarbi et al., 2015; Puga et al., 2017), no new case of HTLV infection was detected after a 1-year follow-up. This finding may have been due to a low occurrence of risk behavior reported by participants. It is important to emphasize that this is the first investigation of new cases of HTLV infection among Brazilian prison inmates after a 1-year follow-up. Despite no new case of HTLV infection, the occurrence of new cases of HCV and HIV, which share the same transmission routes as HTLV, highlights the importance of effective HTLV preventive measures.

This study has limitations that should be considered. Regarding the veracity of the participant’s responses and the possibility of some behavior risks that may have been under-reported due to discrimination and stigma, leading to underestimating potential risk factors associated with HTLV infection. Despite these limitations, our study, having a large sample size, has results that would contribute to a clearer scenario of the circulation of HTLV-1/2 in the inmate population. It is important to emphasize that HTLV infection carriers are potential disseminators, although most of them remain asymptomatic.

Cross-sectional and cohort surveys are important to study the dynamics of this retrovirus in low prevalence areas. This study indicates the circulation of HTLV-1/2 and the concomitant infections by HIV and HCV in the prison population of Mato Grosso do Sul, Central Brazil, representing a serious reality to be considered by the Public Health authorities.

## Data Availability Statement

The original contributions presented in the study are included in the article/supplementary material, further inquiries can be directed to the corresponding author.

## Ethics Statement

The studies involving human participants were reviewed and approved by Federal University of Mato Grosso do Sul Ethics Committee on Human Research Protocol number 1.250.132, CAAE: 49361715.8.0000.0021. The patients/participants provided their written informed consent to participate in this study.

## Author Contributions

LMB, JC, and ARCM-C: conceptualization. LMB, MAMP, JC, MAP, and ARCM-C: data curation. LMB, MAMP, CA, and ARCM-C: formal analysis. JC and ARCM-C: funding acquisition. LMB, MAMP, CA, GRR, MAP, and ARCM-C: investigation and methodology. LMB and ARCM-C: project administration. LMB, MAMP, CA, GRR, JC, MAP, and ARCM-C: writing – original draft and writing – review and editing. All authors contributed to the article and approved the submitted version.

## Conflict of Interest

The authors declare that the research was conducted in the absence of any commercial or financial relationships that could be construed as a potential conflict of interest.

## Publisher’s Note

All claims expressed in this article are solely those of the authors and do not necessarily represent those of their affiliated organizations, or those of the publisher, the editors and the reviewers. Any product that may be evaluated in this article, or claim that may be made by its manufacturer, is not guaranteed or endorsed by the publisher.
